# Radiation dose and survival of patients with stage IV non-small cell lung cancer undergoing concurrent chemotherapy and thoracic three-dimensional radiotherapy: reanalysis of the findings of a single-center prospective study

**DOI:** 10.1186/1471-2407-14-491

**Published:** 2014-07-08

**Authors:** Wei-Wei Ouyang, Sheng-Fa Su, Yin-Xiang Hu, Bing Lu, Zhu Ma, Qing-Song Li, Hui-Qin Li, Yi-Chao Geng

**Affiliations:** 1 Department of Thoracic Oncology, Affiliated Hospital of Guiyang Medical College, Guizhou, Guiyang, China; 2 Guizhou Cancer Hospital, 1 Beijing Road West, Guizhou, Guiyang, China; 3 Teaching and Research Section of Oncology, Guiyang Medical College, Guizhou, Guiyang, China

**Keywords:** Non-small cell lung cancer, Stage IV, Concurrent chemoradiotherapy, Thoracic three-dimensional radiotherapy, Dose, Response

## Abstract

**Background:**

The objective of this study was to evaluate the radiation dose and response in terms of local-regional progression-free survival (LRPFS) and overall survival (OS) of patients with stage IV non-small cell lung cancer (NSCLC) undergoing concurrent chemotherapy and thoracic three-dimensional radiotherapy.

**Methods:**

In all, we enrolled 201 patients with stage IV NSCLC in this study and analyzed OS in 159 patients and LRPFS in 120.

**Results:**

The 1-, 2-, 3-, and 5-year OS rates were 46.2%, 19.5%, 11.7%, and 5.8%, respectively, the median survival time being 12 months. The median survival times in differential treatment response of primary tumors were 19 of complete response, 13 of partial response, 8 of stable disease, and 6 months of progressive disease, respectively (*P* = 0.000). The 1-, 2-, 3-, and 5-year LRPFS rates of patients undergoing four to five cycles with doses ≥63 Gy and <63 Gy were 77.4% and 32.6%, 36.2% and 21.7%, 27.2% and 0, and 15.9% and 0, respectively (*P* = 0.002). According to multivariate analyses, four to five cycles of chemotherapy, gross tumor volume <175.00 cm^3^ and post-treatment Karnofsky Performance Status score stable or increased by at least 10 units were independent prognostic factors for better OS (*P* = 0.035, *P* = 0.008, and *P* = 0.000, respectively). Radiation dose to the primary tumor ≥63 Gy resulted in better OS (*P =* 0.057) and LRPFS (*P =* 0.051), both findings being of borderline significance.

**Conclusions:**

Treatment of IV NSCLC with joint administration of four to five cycles of chemotherapy and three-dimensional radiotherapy may prolong survival, particularly in patients receiving ≥63 Gy radiotherapy, with gross tumor volume <175.00 cm^3^ and post-treatment Karnofsky Performance Status score not lower than pretreatment values.

## Background

Approximately 30% to 40% of patients with non-small cell lung cancer (NSCLC) have stage IV disease at the time of initial diagnosis. Systemic chemotherapy is the standard treatment option for these patients [1,2]. NSCLC patients with epidermal growth factor receptor gene mutations may also receive molecular targeted therapy, which prolongs progression-free survival (PFS) in 20%–30% of advanced cases [3-6]. Approximately 70% of patients still require platinum-based combination chemotherapy; different third-generation chemotherapy regimens have similar efficacy, indicating that the efficacy of chemotherapy has reached a plateau [7]. The efficacy of palliative two-dimensional (2D) radiotherapy for treating stage IV NSCLC patients with thoracic lesions has been systematically reviewed; the reviewers concluded that there were flaws in some aspects of the designs of the studies they reviewed. Therefore, they questioned the validity of the conclusions drawn in those studies [8]. Three-dimensional (3D) radiotherapy is another treatment option for patients with NSCLC [9]. The results of Radiation Therapy Oncology Group 0617, which randomly assigned patients to radiation doses of 60 Gy versus 74 concurrently with carboplatin and paclitaxel, failed to show any benefit for higher radiation dose in patients with locally advanced NSCLC [10]. In contrast, the response rate and higher dose correlated strongly with improved overall survival (OS) and/or PFS in patients with locally advanced NSCLC [11,12]. In advanced NSCLC, a higher dose correlated with improved survival in the studies of Su *et al*. and Lopez *et al*. [13,14]. The present study was designed to evaluate the associations between local radiation dose, local response, and survival in stage IV NSCLC patients undergoing concurrent chemotherapy and thoracic 3D radiotherapy.

## Methods

### Inclusion and exclusion criteria

This prospective study was performed with the approval of the Institutional Review Board of the Affiliated Hospital of Guiyang Medical College, and Guizhou Cancer Hospital China and informed consent was obtained from all patients. The inclusion criteria were as follows: 1) histologically or cytologically confirmed NSCLC; 2) newly diagnosed stage IV disease according to the staging system of the 2002 American Joint Committee on Cancer; 3) age 18–80 years with a Karnofsky Performance Status (KPS) score ≥70; 4) limited metastatic disease (≤five sites); 5) no contraindications to radiotherapy and chemotherapy; 6) underwent at least two cycles of chemotherapy and received radiation at doses ≥36 Gy; and 7) adequate bone marrow, liver, and renal function (defined as neutrophil count ≥1.5 × 10^9^/L, platelet count 80 × 10^9^/L, hemoglobin concentration ≥80 g/L, aspartate aminotransferase and alanine aminotransferase concentrations ≤2× the upper limit of the institutional normal range, total bilirubin ≤1.25× the upper limit of the institutional normal range, and creatinine concentration ≤120 μmol/L). The exclusion criteria were as follows: 1) previous thoracic surgery, radiotherapy, or chemotherapy; 2) malignant pleural or pericardial effusion; 3) pregnancy or lactation; and 4) previous malignancy or other concomitant malignant disease.

### Pre- and post-treatment assessment

Means of pretreatment assessment comprised physical examination, hematologic and biochemical profiles, fiberoptic bronchoscopy, contrast-enhanced computed tomography (CT) scanning of the chest and abdominal region, plain or contrast-enhanced magnetic resonance imaging (MRI) of the head, and bone scintigraphy. Additional investigations were performed if indicated. All the same assessments, except for fiberoptic bronchoscopy and bone scintigraphy, were also performed after therapy.

### General clinical data

In all, 201 patients diagnosed with stage IV NSCLC between January 2003 and July 2010 were enrolled in this study. Nineteen patients underwent only one cycle of chemotherapy, 10 received <36 Gy and 13 cases refused post-treatment evaluation by imaging. These 42 cases were omitted from the analyses. Combination chemotherapy and radiation were administered to intrathoracic primary tumors in the remaining 159 patients, 119 of whom were male and 40 female. The patients were aged 30–80 years (median age 59 years). The median gross tumor volume (GTV) was 175.00 cm^3^ (16.55–892.00 cm^3^). The median number of cycles of chemotherapy was 4 (2–5 cycles), and the median dose to the planning target volume was 63 Gy (36–72 Gy). Post-treatment progression of the primary tumor occurred in 11 patients. Twenty-eight patients participated in post-treatment follow-up via telephone but refused re-examination by imaging; thus, 120 were assessed for local-regional progression-free survival (LRPFS). These patients’ clinical characteristics are listed in Table 1. Significantly more patients received four to five cycles with radiation doses ≥ 63 Gy than <63 Gy. Metastases were in bone, lung, brain, liver, renal capsule and other sites. The most common sites of metastatic disease were bone (52.2%), lung (36.5%), and brain (22.6%). Metastases were detected in only one site in 98 cases (61.6%), in two sites in 45 cases (28.3%), in three sites in 12 cases, four sites in three cases, and five sites in one case.

**Table 1:** **Clinical characteristics of patients (*n*** **= 120) in whom local-regional progression-free survival was assessed**

**Variable**	**<63 Gy**	**≥63 Gy**	***χ***^***2***^	***p***
Gender				
Male	33	53	2.387	0.122
Female	8	26		
Age (years)				
<65	27	55	0.177	0.674
≥65	14	24		
Pathological type				
Squamous	17	23	5.268	0.072
Adenocarcinoma	18	51		
Other types	6	5		
T stage				
T1	3	7	2.371	0.499
T2	17	23		
T3	6	10		
T4	15	39		
N stage				
N0	3	5	2.631^#^	0.444
N1	2	10		
N2	14	31		
N3	22	33		
GTV (cm^3^)	23.64 ~ 892.00 (204.91)	16.55 ~ 617.50 (160.51)	1.108*	0.171
Chemotherapy				
2-3 cycles	23	23	8.314	0.004
4-5 cycles	18	56		
Range (median)	2-4 (3)	2-5 (4)		
Organ metastases				
Single organ	27	54	0.077	0.781
Multi-organ	14	25		
Treatment response of primary tumor				
CR	1	10	3.961	0.138
PR	32	59		
NC	8	10		

Note: *Student *t-*test; GTV from a minimum–maximal value (median); ^#^Fisher’s exact test.

### Chemotherapy protocol

Platinum-based doublets chemotherapy was administered according to the following regimens: 80 mg/m^2^ cisplatin (C) or carboplatin (Cb; 6 AUC) intravenously on day 2, 140 mg/m^2^ paclitaxel (P) or 75 mg/m^2^ docetaxel (D) on day 1, and 25 mg/m^2^ vinorelbine (V) on days 1 and 8 every 21–28 days during thoracic radiotherapy. Concurrent thoracic radiation was given within 1 week of starting chemotherapy. After completion of thoracic radiotherapy, patients with a response or stable disease continued chemotherapy for up to four to six cycles, whereas second-line therapy was substituted in patients with progressive disease or unacceptable toxicity. PC or PCb combination chemotherapy was administered to 40.3% of patients (64/159), DC or DCb to 53.4% (85/159), and VC to 6.3% (10/159). Thirty-one, 41, 83, and four patients underwent two, three, four, and five cycles of chemotherapy, respectively. In all, 537 cycles were administered.

### Radiotherapy protocol

The treatment was planned using ADAC Pinnacle^3^ radiation treatment planning software (version 7.4f). The GTV encompassed both the primary lung lesion and any hilar and mediastinal lymph nodes that were visible on the treatment planning CT scan. The GTV plus a margin of 15 mm was defined as the planning target volume, the area covered by at least a 90% isodose surface. The V20 (percentage of total lung volume receiving ≥20 Gy), maximum point dose to the spinal cord and mean esophageal dose were required to be ≤32%, 50 Gy and ≤35 Gy, respectively, in each individual treatment plan. Patients received late-course accelerated hyperfractionated radiotherapy to the thoracic primary site using 3D-conformal or intensity-modulated radiation therapy. The first course of radiotherapy was given in two Gy fractions for 5 days a week to a total dose of 36–40 Gy, whereas late-course accelerated hyperfractionated radiotherapy was delivered in two fractions of 1.5 Gy each with an interval of 6–8 h each day. The protocol required delivery of a prescription dose of 60–70 Gy to patients; individuals who did not tolerate this were to receive ≥36 Gy. Provided radiation doses to normal tissue were acceptable, the radiation dose to the primary thoracic tumor could be escalated to 72 Gy. Sixty-seven patients received <63 Gy and 92 ≥ 63 Gy (70 received 63 Gy and 22 received 63–72 Gy). Metastatic lesions were treated with 3D-conformal radiation therapy or 2D hypofractionated radiotherapy. In all, 98 patients received radiotherapy concurrently or sequentially with chemotherapy for metastatic lesions in 3–10 Gy daily fractions to a total dose of 20–60 Gy.

### Evaluation of therapeutic efficacy

The responses of the primary tumors, including complete response (CR), partial response (PR), stable disease (SD), and progressive disease (PD), were evaluated according to the World Health Organization response criteria. LRPFS was defined as the length of time between the start of treatment and either progression of the primary tumor or death. A comprehensive evaluation of both the primary tumor and distant metastases was performed 1 month after completion of chemoradiotherapy treatment. For primary tumors and metastatic lesions other than those in bone, response rate was defined as CR or PR. However, for patients with bone metastases, SD (i.e. no evidence of PD) of bone metastases was also regarded as a response. Nonresponse of primary tumors was defined as larger and/or stable lesions and of metastatic tumors as SD, PD, or development of new metastatic lesions.

### Follow-up and statistical analyses

After completion of treatment, the patients underwent contrast-enhanced CT scanning of the chest and abdominal region and MRI of the head every 3 months for 2 years, and every 6 months thereafter. Bone scintigraphy was performed every 6 months for 2 years, and every 12 months thereafter. All statistical analyses were performed using the Statistical Package for Social Sciences version 13.0 (SPSS; Chicago, IL, USA). The significance of differences in proportions was assessed with the *χ*^2^ test. Kaplan–Meier analyses were performed to estimate LRPFS and OS and the log-rank test to compare the survival curves. The Cox proportional hazards model was used to perform multivariate analyses to assess the LRPFS and OS. Results were considered statistically significant when the two-tailed *P* value was <0.05.

## Results

### Differential treatment response of primary tumors

Follow-up ended on 1 March 2013. Four patients were lost to follow-up; the remaining 155 patients (97.5%) underwent follow-up for 2–80 months (median 12 months). One hundred and forty-five patients had died by the study end-point, 34 (23.5%) from local progression, 26 (17.9%) from local progression and distant metastases, and 85 (58.6%) from distant metastases alone. Ten patients were still alive, their survival times being 34–80 months. The 1-, 2-, 3-, and 5-year OS rates of the 159 study patients were 46.2%, 19.5%, 11.7%, and 5.8%, respectively; their median survival time (MST) was 12 (95% CI, 9.8–14.2) months. Twelve/159 patients (7.5%) achieved CR, 66.0% (105/159) PR, 19.5% (31/159) SD, and 6.9% (11/159) PD, the MSTs being 19 (95% CI, 8.5–29.5), 13 (95% CI, 10.9–15.1), 8 (95% CI, 6.4–9.6), and 6 (95% CI, 4.4–7.6) months, respectively (*χ*^2^ = 24.330, *P* = 0.000). The 1-, 2-, 3-, and 5-year OS rates of patients with CR/PR and SD/PD were 54.4% and 23.8%, 22.3% and 11.9%, 13.4% and 7.1%, and 5.4% and 4.8%, respectively, their MSTs being 14 (95% CI, 11.9–16.1) and 7 (95% CI, 5.4–8.6) months, respectively (*χ*^*2*^ = 8.345, *P* = 0.004).

### Comparison of LRPFS according to treatment responses of primary tumors

The median LRPFS of the 120 patients in whom it was assessed was 16 months (95% CI, 12.5–19.5). The 1-, 2-, 3-, and 5-year LRPFS rates were 57.5%, 30.5%, 21.0%, and 10.2%, respectively. The LRPFS of patients in CR seemed to be longer than of those in PR and SD; however, these differences were not significant (Table 2).

**Table 2:** Local-regional progression-free survival in 120 stage IV NSCLC patients according to responses of primary tumors

**Variable**	**No.**	**Survival rate (%)**				**Median survival time (months)**	**95% confidence interval**		***χ***^***2***^	***p***
		**1 year**	**2 year**	**3 year**	**5 year**					
CR	11	63.6	42.4	31.8	31.8	20	8.5	31.5	2.065	0.356
PR	91	58.3	27.5	18.3	0	15	11.5	18.5		
SD	18	50.0	35.7	26.8	26.8	10	1.9	18.1		
CR	11	63.6	42.4	31.8	31.8	20	8.5	31.5	1.922	0.166
PR + SD	109	56.9	29.0	19.7	7.8	15	11.9	18.1		

### LRPFS according to radiation doses to primary tumors

**Figure 1:**
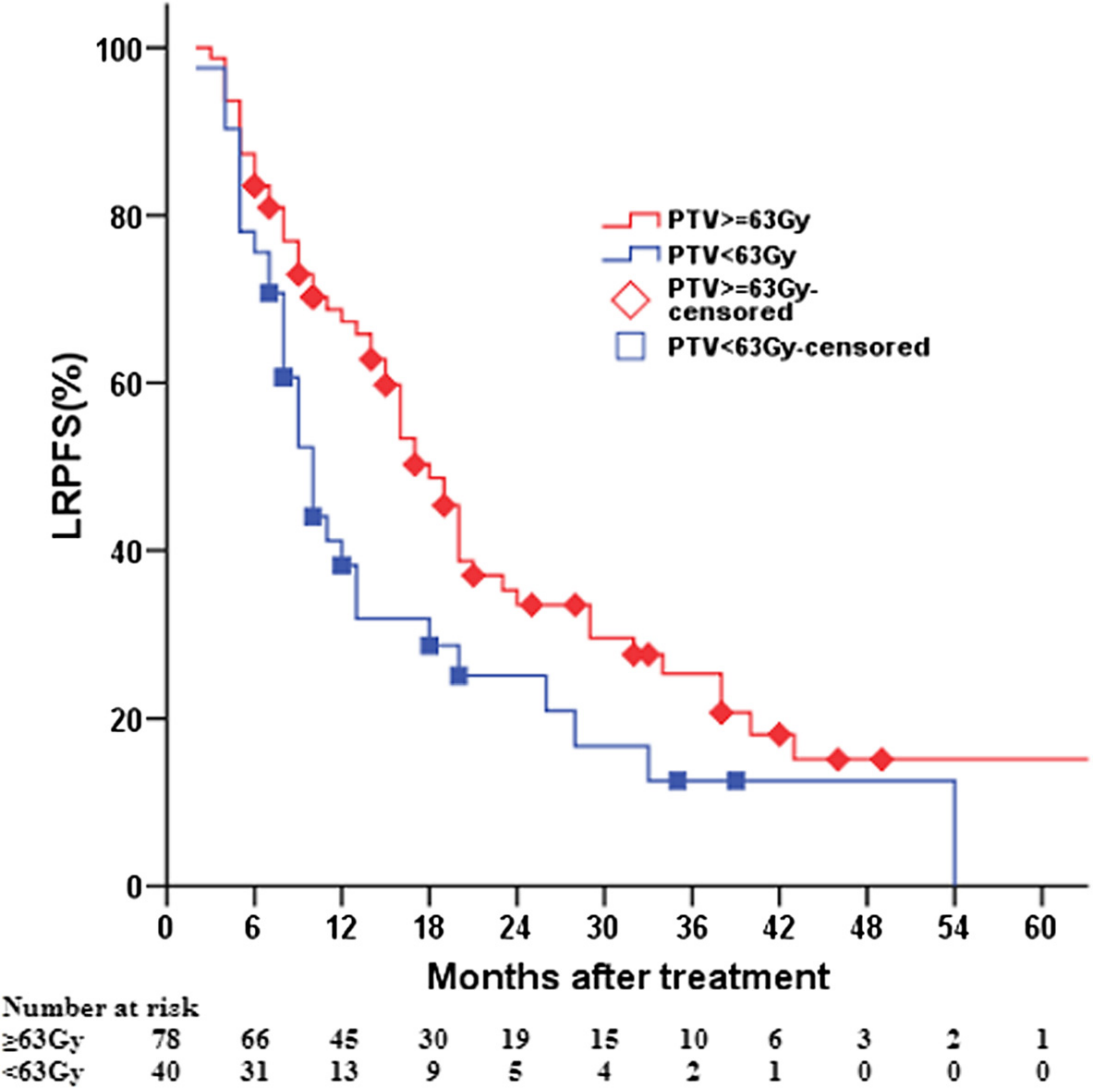
Comparison of local-regional progression-free survival according to radiation dose to primary tumors.

The 1-, 2-, 3-, and 5-year LRPFS rates of patients receiving radiation doses of ≥63 Gy or <63 Gy were 67.3% and 38.2%, 33.5% and 25.1%, 25.3% and 12.5%, and 15.1% and 0, respectively, their MSTs being 18 (95% CI, 15.2–20.8) and 10 (95% CI, 8.1–11.9) months, respectively (*χ*^*2*^ = 5.458, *P* = 0.019) (Figure 1).

### Assessment of LRPFS in patients who received concurrent chemotherapy and various doses of radiation to primary tumors

The 1-, 2-, 3-, and 5-year LRPFS rates of patients undergoing four to five cycles with doses ≥63 Gy versus <63 Gy were 77.4% and 32.6%, 36.2% and 21.7%, 27.2% and 0, and 15.9% and 0, respectively, their MSTs being 20 (95% CI, 16.5–23.5) and 9 (95% CI, 6.6–11.4) months, respectively (*χ*^*2*^ = 9.217, *P* = 0.002) (Table 3). The difference between patients undergoing two to three cycles with doses ≥63 Gy versus <63 Gy (*χ*^*2*^ = 0.040, *P* = 0.842) was not significant.

**Table 3:** Local-regional progression-free survival in 120 stage IV NSCLC patients according to radiotherapy dose

**Cycle**	**Dose (Gy)**	**No.**	**Survival rate (%)**				**Median survival (months)**	**95% confidence interval**		***χ***^***2***^	***p***
			**1 year**	**2 year**	**3 year**	**5 year**					
2-3	<63	23	41.7	27.8	20.9	0	10	5.5	14.5	0.040	0.842
	≥63	23	43.5	26.1	19.6	0	9	4.3	13.7		
4-5	<63	18	32.6	21.7	0	0	9	6.6	11.4	9.217	0.002
	≥63	56	77.4	36.2	27.2	15.9	20	16.5	23.5		

### Effect of various combined chemoradiotherapy regimens on LRPFS and OS

**Figure 2:**
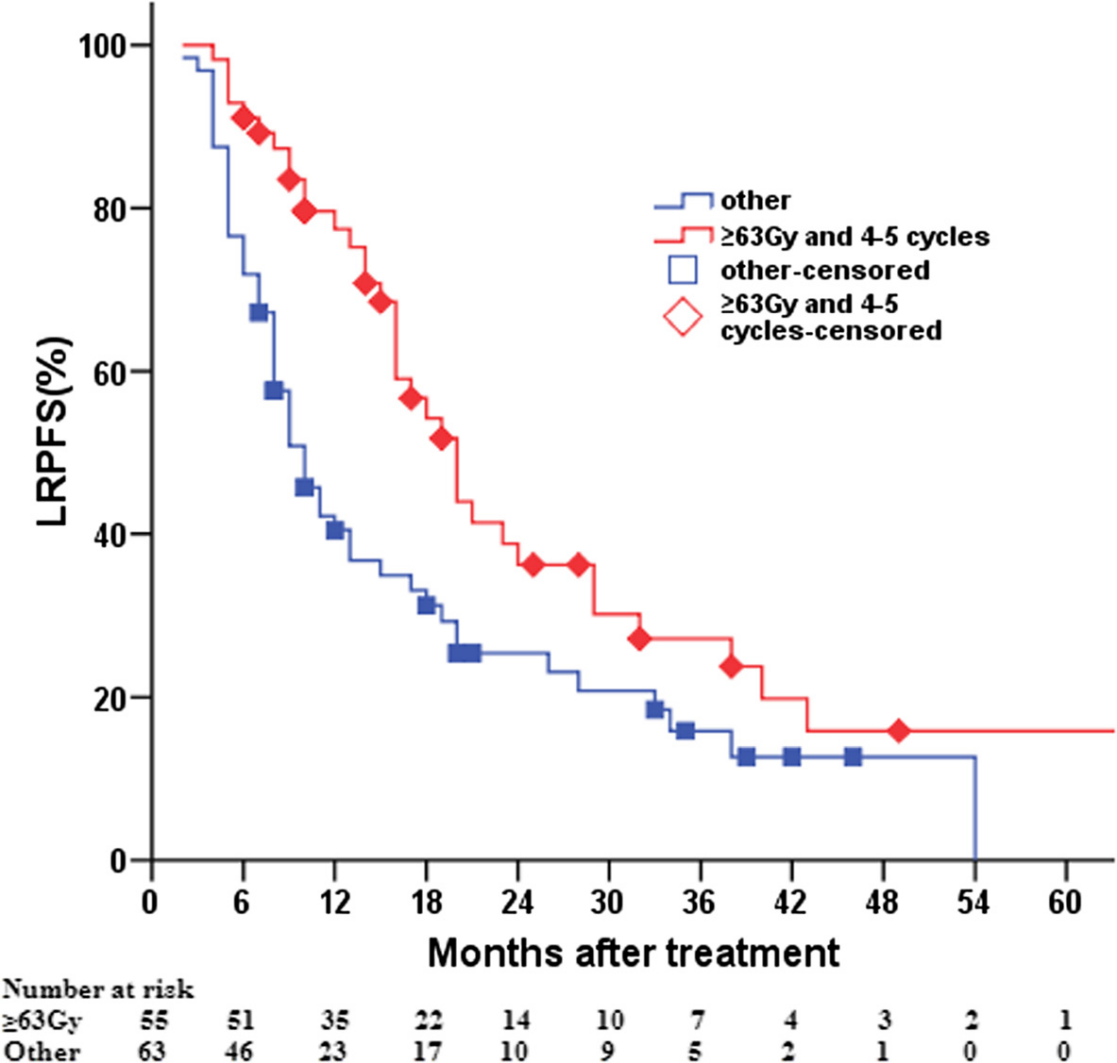
Comparison of local-regional progression-free survival between patients who underwent four to five cycles of chemotherapy concurrently with radiotherapy at doses of ≥63 Gy and patients who received other treatment regimens.

One hundred and five cases achieved responses and 54 did not. The 1-, 2-, 3-, and 5-year OS rates of patients who did and did not respond were 60.7% and 18.5%, 24.9% and 9.3%, 14.9% and 5.6%, and 6.1% and 3.7%, respectively, their MSTs being 15 (95% CI, 12.9–17.1) and 7 (95% CI, 6.0–8.0) months, respectively (*χ*^*2*^ = 20.715, *P* = 0.000). The 1-, 2-, 3-, and 5-year LRPFS rates of patients who received four to five cycles with doses ≥63 Gy and two to three cycles with doses ≥63 Gy were 77.4% and 43.5%, 36.2% and 26.1%, 27.2% and 19.6%, and 15.9% and 0, respectively, their MSTs being 20 (95% CI, 16.5–23.5) and 9 (95% CI, 4.3–13.7) months, respectively (*χ*^*2*^ = 3.108, *P* = 0.078). In addition, the 1-, 2-, 3-, and 5-year LRPFS rates of patients who received four to five cycles with doses ≥63 Gy compared with other treatment regimens (including two to three cycles with doses ≥63 Gy, two to three cycles with doses <63 Gy, and four to five cycles with doses <63 Gy) were 77.4% and 40.5%, 36.2% and 25.4%, 27.2% and 15.8%, and 15.9% and 0, respectively, their MSTs being 20 (95% CI, 16.5–23.5) and 10 (95% CI, 7.6–12.4) months, respectively (*χ*^*2*^ = 8.065, *P* = 0.005) (Figure 2).

### Cox regression analyses

According to multivariate analyses, patients who received four to five cycles of chemotherapy (HR, 1.484; 95% CI, 1.028–2.144; *P* = 0.035), GTV <175.00 cm^3^ (HR, 1.747; 95% CI, 1.153–2.646; *P* = 0.008) and post-treatment KPS score stable or increased by at least 10 units (HR, 6.282; 95% CI, 3.568–11.058; *P* = 0.000) were independent predictors of better OS. Both N stage (HR, 1.636; 95% CI, 0.981–2.727; *P =* 0.059) and the primary tumor radiation dose (HR, 1.433; 95% CI, 0.989–2.075; *P =* 0.057) were of borderline significance with respect to OS. GTV <175.00 cm^3^ (HR, 2.321; 95% CI, 1.336–4.032; *P* = 0.003) and post-treatment KPS score (HR, 8.038; 95% CI, 3.769–17.140; *P* = 0.000) were independent predictors of LRPFS. The primary tumor radiation dose was of only borderline significance with respect to LRPFS (HR, 1.562; 95% CI, 0.999–2.443; *P =* 0.051) (Table 4).

**Table 4:** Multivariate analysis: overall survival and local-regional progression-free survival

**Variable**	**OS**			**LRPFS**		
	***p***	**HR**	**95% CI**	***p***	**HR**	**95% CI**
Gender (Female vs. Male)	0.288	0.802	0.534-1.205	0.278	0.753	0.450-1.258
Age (years) (≥65 vs. <65)	0.297	0.817	0.558-1.195	0.248	0.760	0.477-1.211
Pathological type (Non-squamous vs. Squamous)	0.106	1.364	0.936-1.988	0.494	1.184	0.729-1.924
T stage (T3-T4 vs. T1-T2)	0.728	1.067	0.740-1.540	0.702	1.097	0.684-1.759
Distant metastatic lesions (No radiotherapy vs. Radiotherapy)	0.682	1.083	0.740-1.584	0.652	1.118	0.689-1.812
Intrathoracic primary tumors (No response vs. Response)	0.592	1.124	0.733-1.724	0.581	1.188	0.644-2.190
GTV (<175 cm^3^ vs. ≥175 cm^3^)	0.008	1.747	1.153-2.646	0.003	2.321	1.336-4.032
Post-treatment KPS (Decreased ≥10 vs. Stable or increased ≥10)	0.000	6.282	3.568-11.058	0.000	8.038	3.769-17.140
N stage (N2-N3vs. N0-N1)	0.059	1.636	0.981-2.727	0.234	1.438	0.790-2.615
Chemotherapy cycles (2–3 vs. 4–5)	0.035	1.484	1.028-2.144	0.128	1.431	0.903-2.268
Intrathoracic primary tumors radiation doses (<63Gy vs. ≥63Gy)	0.057	1.433	0.989-2.075	0.051	1.562	0.999-2.443

## Discussion

In patients with stage IV NSCLC, four to six cycles of platinum-based combination chemotherapy typically results in 1-year survival rates of approximately 30%–45%, median time to progression of 3–5 months, and median survival time of 8–10 months [1,7,15-17]. However, uncontrolled primary tumor progression, which adversely affects survival, occurs in approximately 50% of all patients [18]. Higginson *et al*. [19] performed a pooled analysis of 189 patients enrolled at a single institution. They found that pulmonary symptoms, total volume of intrathoracic lesions, and bronchial/vascular compression all influenced survival after first-line, platinum-based chemotherapy. A subset of these patients could be studied to determine whether early, planned palliative thoracic radiation would be beneficial. Whether palliative 2D radiotherapy for chest lesions prolongs patient survival is unknown and debatable [20,21]. Several prospective clinical trials have been reviewed and found to have had flaws in the enrollment of patients undergoing radiotherapy alone; these flaws likely skewed the interpretation of the effects of radiotherapy [8]. Future research should focus on testing multiple regimens of combined chemotherapy (typically four to six cycles) and 3D radiotherapy for primary tumors with stage IV NSCLC patients. An earlier retrospective study showed that patients with KPS scores ≥90 who underwent stereotactic radiotherapy or surgical excision of solitary cerebral metastatic lesions concurrently with radical chemoradiotherapy or surgery for intrathoracic lesions had a significantly longer MST and higher survival rates than those whose intrathoracic lesions were not treated (*P* = 0.000) [22], indicating that patients with few metastases may benefit from radical therapy for intrathoracic lesions [14,18]. In this study, the OS time of patients who responded to treatment was significantly longer than of those who did not respond; the LRPFS of patients with CR was 5 months longer than of those with PR/SD. These data suggest that the survival of patients with stage IV NSCLC may be improved by administering local radiotherapy to their primary tumors. An earlier prospective trial showed that a 30 Gy/10 fractions radiotherapy schedule is better than a 16 Gy/2 fractions schedule for palliative treatment of patients with stage IV NSCLC [23]. A systematic review of 13 randomized controlled trials of palliative thoracic radiotherapy showed that metastases are the major factor resulting in failure of treatment of stage IV NSCLC; the presence of uncontrolled intrathoracic lesions also significantly affects treatment failure. High dose (35 Gy_10_ BED) palliative thoracic radiotherapy reportedly improves the total symptom score and 1-year overall survival compared with lower dose radiotherapy. These data indicate that some patients benefit from receiving local radiotherapy [24]. A systematic review has shown that increasing fractionation and total radiation dose to primary tumors may prolong patient survival [25]. Thus, further prospective clinical trials to evaluate the efficacy of modern 3D radiotherapy given concurrently with combination chemotherapy for stage IV NSCLC are warranted. A long-term study in which the radiation dose was escalated found that increasing the dose improved the control rate of local tumors in patients with locally advanced NSCLC [12]. In our study, the LRPFS rate of patients who received intrathoracic radiotherapy at doses ≥63 Gy was significantly higher than that of patients who received <63 Gy. Importantly, four to six cycles of chemotherapy has a significantly greater effect on survival than does two to three cycles of chemotherapy [26]. In the current study, patients who received intrathoracic radiation at doses ≥63 Gy underwent a median of four cycles of chemotherapy, which was one more cycle than was given to patients who received radiation at doses <63 Gy. Whether the intensity of chemotherapy affects the outcome remains unclear. Subset analysis showed that the LRPFS rate of patients who underwent two to three cycles of chemotherapy concurrently with radiation at doses of ≥63 Gy was similar to that of patients who underwent two to three cycles of chemotherapy concurrently with radiation at doses of <63 Gy. In contrast, the LRPFS rate was significantly higher in patients undergoing four to five cycles of chemotherapy concurrently with radiotherapy at doses of ≥63 Gy. These data are consistent with findings from an earlier retrospective study of 78 consecutive patients with oligometastatic NSCLC. That study found that patients who received at least 63 Gy of radiation to their primary tumors had better OS, PFS, and local control rate than those who received <63 Gy, suggesting that controlling metastases by chemotherapy is equivalent to achieving local control with 3D radiotherapy in such patients [14]. The most efficient therapeutic strategy for stage IV NSCLC involves both control of the primary tumor and treatment of metastatic lesions. In this study, the LRPFS was longer in patients who received at least 63 Gy of radiation to their primary tumors concurrently with four to five cycles of chemotherapy than in those who underwent other treatment regimens. Multivariate analysis showed that four to five cycles of chemotherapy is a predictor of better OS. A primary tumor radiation dose ≥63 Gy provided better OS and LRPFS, these findings being of borderline significance. Thus, there is a trend for thoracic 3D radiotherapy together with systemic chemotherapy to prolong survival of patients with stage IV NSCLC.

The prognosis of NSCLC patients is closely associated with both pre-treatment [9] and post-treatment performance status. In the present study, the patients whose post-treatment KPS scores were stable or increased had higher OS rates and longer LRPFS than patients whose scores decreased. This suggests that post-treatment performance status should be maintained or improved; thus, overtreatment should be avoided when treating stage IV NSCLC with integrated treatment regimens.

## Conclusions

Four to five cycles of chemotherapy given concurrently with 3D radiotherapy for intrathoracic tumors may prolong survival, particularly in patients who receive radiotherapy at doses ≥63 Gy, GTV < 175.00 cm^3^ and whose post-treatment KPS scores do not decrease.

## Abbreviations

NSCLC: Non-small cell lung cancer; LRPFS: Local-regional progression-free survival; OS: Overall survival; PFS: Progression-free survival; MRI: Magnetic resonance imaging; CT: Computed tomography; 2D: Two-dimensional; 3D: Three-dimensional; KPS: Karnofsky Performance Status; GTV: Gross tumor volume; C: Cisplatin; Cb: Carboplatin; P: Paclitaxel; D: Docetaxel; V: Vinorelbine; MST: Median survival time.

## Competing interests

The authors declare that they have no competing interests.

## Authors’ contributions

BL designed the study. W-WO, S-FS, Y-XH, ZM, Q-SLi, H-QLi, and Y-CG collected the data. W-WO, S-FS, Y-XH, and BL undertook the data analyses and interpretation, performed the statistical analyses and wrote the report. All authors have read and approved the final manuscript.

## Pre-publication history

The pre-publication history for this paper can be accessed here:

http://www.biomedcentral.com/1471-2407/14/491/prepub
